# Ill-Nature Rebuked

**Published:** 1873-11

**Authors:** 


### ILL-NATURE REBUKED.

A GREAT many years ago, when I was a little girl, I started to take a journey to see my aunt—not in the cars—they had never thought of such a thing then—but in the stage. Now I felt very proud to be going away off without papa, or mamma, or nurse to take care of me, and only my Uncle Charlie alone, who was a gay, pleasant young man in college. Now I sat snugly tucked beside uncle on the back seat, sitting very straight, and wondering very much in my silly little heart if the gentleman on the front-seat would not think I was a young lady—father said I was so large of my age—and then more silly, may be he would think I was grown up, and was Uncle Charlie's wife. Oh! how absurd it was, was it not, children, that I, only nine years old, should have ever thought of such a thing? My grown-up consequential feelings did not last long, though, for soon the stage stopped, and a very feeble-looking old man with a little girl, whose hood covered up her whole face, got in. The old man saw Uncle Charlie's pleasant face, and said: "If you please, sir, take your little girl on your lap, and I will mine. I like to ride on the back-seat, the others make me sick." "Certainly, sir." And my dignity was very summarily disposed of, by uncle's lifting me, without another word, into his lap. He only laughed, because he had no little girl, and it was a funny mistake. But I did not laugh. I pouted and made uncle very uncomfortable with my fidgeting about, and sour, hateful looks. I happened to look up in a few minutes, and I saw the child, sitting on the old man's lap, had her hood taken off, but her eyes all covered up with a great, thick bandage. Soon she spoke in the sweetest voice to the old man: "Grandpa, may be we could sit somewhere else, and let the little girl sit here." How I wonder that she knew I was cross about it, with her eyes all covered up, so she could not see my face, and I had been ashamed to say any thing. "Oh! no," said I, sorry, and forgetting my ill-humor in wondering why she kept her eyes covered all up that way.

Again I said: "Please don't be hurt at me, but won't you tell me what ails your eyes?" "Oh! yes," she said, very sweetly, "I was coming down-stairs with the scissors in my hands, and I put my eye out, and then the other got blind, too, and now I can never see out of either any more. But I am going to Boston to try and have the doctor there do something for them, so that they won't hurt me so badly." My eyes filled with tears for the poor blind girl. "Can she never see again?" said my uncle. "No, there is no hope of that," said the old man very sadly. "Grandpa says I can see when I get to heaven," said she in a very low whisper, and looking very cheerful and bright as she said it. "Is she happy that way, always?" said my uncle. "Yes, always. Every one calls her 'happy Mary.'"

She got out soon, said grandma—taking off her spectacles, and even then wiping her eyes—and I never saw her again, but I never forgot her; but I always remembered, when I was inclined to be cross over little things, poor blind Mary, who would never see till she got to heaven; and yet whom every one called "happy Mary."—*Western Churchman.*

